# Synaptic transistors with aluminum oxide dielectrics enabling full audio frequency range signal processing

**DOI:** 10.1038/s41598-020-73705-w

**Published:** 2020-10-07

**Authors:** Sami Bolat, Galo Torres Sevilla, Alessio Mancinelli, Evgeniia Gilshtein, Jordi Sastre, Antonio Cabas Vidani, Dominik Bachmann, Ivan Shorubalko, Danick Briand, Ayodhya N. Tiwari, Yaroslav E. Romanyuk

**Affiliations:** 1 Empa-Swiss Federal Laboratories for Materials Science and Technology, Ueberlandstrasse 129, 8600 Dübendorf, Switzerland; 2 Ecole Polytechnique Fédérale de Lausanne (EPFL), Soft Transducers Laboratory LMTS, Neuchâtel, Switzerland

**Keywords:** Bioinspired materials, Electronic devices, Synaptic plasticity, Electronic and spintronic devices

## Abstract

The rapid evolution of the neuromorphic computing stimulates the search for novel brain-inspired electronic devices. Synaptic transistors are three-terminal devices that can mimic the chemical synapses while consuming low power, whereby an insulating dielectric layer physically separates output and input signals from each other. Appropriate choice of the dielectric is crucial in achieving a wide range of operation frequencies in these devices. Here we report synaptic transistors with printed aluminum oxide dielectrics, improving the operation frequency of solution-processed synaptic transistors by almost two orders of magnitude to 50 kHz. Fabricated devices, yielding synaptic response for all audio frequencies (20 Hz to 20 kHz), are employed in an acoustic response system to show the potential for future research in neuro-acoustic signal processing with printed oxide electronics.

## Introduction

Simulating synapses with electronic devices while minimizing power consumption, chip area, and manufacturing cost is fundamental to future applications of brain-inspired electronics and neuromorphic computing^1^. Implementation of the synapses has been demonstrated so far with two terminal^1^ and three-terminal electron devices^2^. Three-terminal devices, namely synaptic (or neuromorphic) transistors, which can realize both memory and signal processing functions simultaneously, offer efficient synapse simulation^2^. In such devices, a gate contact is used as the presynaptic input, whereas the channel conductance represents the postsynaptic output. The input current can be made several orders of magnitude lower than that of the two-terminal devices since the input is provided through a gated insulating layer, which results in improved memory retention and energy efficiency. Synaptic transistors, when coupled with materials sensitive to different stimuli, can also provide pathways to mimic the behavior of the afferent (sensory) and efferent (motor) neurons.

Synaptic behavior in the transistors have been demonstrated so far with different classes of gate dielectric materials and device architectures, such as floating gate^3^, metal oxide dielectrics^4–6^ electrolyte insulators^7^, ionic liquids^8^, proton conductors^9^, and ferroelectric materials^10^. Among these approaches, electrolyte gating and ionic liquids promise ultra-low voltage (< 1 V) operation. This is enabled by Helmholtz electric double layer capacitance, which is at least one order of magnitude higher than the gate capacitances provided by conventional oxide dielectrics^11^. Due to the increased gate capacitance, devices can turn on at lower voltages enabling low power synapse simulation. The electrolytes can be processed using vacuum, solution- and printing methods^7,12^. Electrolyte gated transistors have been demonstrated to operate at frequencies reaching MHz levels for switching and analog amplification enabled by internal ion gating mechanisms in integrated circuits for bio-electronic applications^12,13^. However, the synaptic operation of electrolyte-gated transistors, which is associated with hysteretic transfer characteristics to provide memory behavior, governed by the ion migration throughout the electrolyte layer, has been demonstrated up to an operating frequency of few kHz^14^. Such a rate is comparable to the millisecond-response time of most synaptic connections in the human neural system but not to the acoustic signal processing that requires the synaptic operation of up to 20 kHz. This limitation disables such devices from mimicking all the chemical synapses in the human neural system. Redox transistors, operating via electrochemical doping of the channel layer by electrolyte dielectric, have been shown to enable synaptic operation at frequencies higher than 1 MHz. Yet, these devices require another switching element for their implementation into the neural networks^15^. Synaptic transistors are advantageous in this respect because they do not need an additional switching element while updating their synaptic weights for their circuit implementations^16^. Ferroelectric field-effect synaptic transistors use spontaneous electric polarization in a solid-state ferroelectric insulator and operate up to megahertz levels^10,14^. However, the manufacturing of the CMOS compatible oxide ferroelectric dielectrics is quite demanding. It includes several steps such as capping with titanium nitride layers and annealing at temperature levels over 450 °C to obtain the desired ferroelectric phase and requires complex lithographic patterning in order not to deteriorate the ferroelectric behavior of the insulator^10,17^ Different gating mechanisms should be investigated to achieve synaptic operations in transistors without complex manufacturing to provide a wide range of operating frequencies.

Therefore, recently, another class of dielectrics, metal oxides, has gained the attraction of the researchers for their implementation in synaptic transistors. Metal oxide dielectrics, per their processing, can possess charge-trapping behavior^4,5^ or can be implemented in floating gate mechanisms^18^, thereby providing memory behavior. These solid-state materials can be deposited at low processing temperatures via vacuum-based deposition methods^4,14^, as well as low-cost solution-based methods^5,6^. If the synaptic operation is enabled for a wide range of operation frequencies, metal oxide dielectrics can fill the gap between the electrolyte gated synaptic devices and ferroelectric transistors in future applications of bio-inspired electronics. Metal oxide dielectrics in synaptic transistors such as AlO_x_ and Gd_2_O_3_^6^ have been produced by atomic layer deposition^4^ or spin coating^5,6^ with subsequent lithographical patterning of the deposited layers, so far^4–6^ and their synaptic operation frequency have been demonstrated up to 500 Hz. Printing offers an advantage of avoiding lithographic patterning of the layers, which is desirable for roll-to-roll manufacturing of low-cost electronic devices and circuits. Up to now, synaptic transistors with printed dielectrics have only been implemented with electrolyte-gated mechanisms operating at frequencies up to 50 Hz^7,19^.

Here we report synaptic transistors with printed aluminum oxide (AlO_x_) dielectrics, which enable an operation frequency of 50 kHz, thus improving the speed of solution-processed synaptic transistors by almost two orders of magnitude. We investigate the structure and composition of printed solid-state AlO_x_ dielectrics to reveal the origins of high-speed memory behavior. Since the synaptic response of the TFTs of this work covers the entire audio frequency range, they can be implemented for neuro-acoustic applications. Neuromorphic processing of sound signals has been shown by very large scale integrated (VLSI) circuit based neural networks as well as field-programmable gate array (FPGA) based neural networks for various tasks, including speech recognition^20,21^, sound identification^22,23^, noise filtering^24^, or sound location detection^25^. A network employing synaptic transistors has also been used for sound location detection by mimicking the spatiotemporal synaptic signal processing, yet input signal frequencies were limited up to 40 Hz^26^. In the present work, we demonstrate the applicability of the synaptic transistors with printed aluminum oxide dielectrics for the acoustic signal processing in the full audio frequency range. A system is shown in which the sound waves received from the environment are converted into square signals with the same frequencies, which serve as presynaptic inputs to oxide-based synaptic transistors.

**Figure 1:**
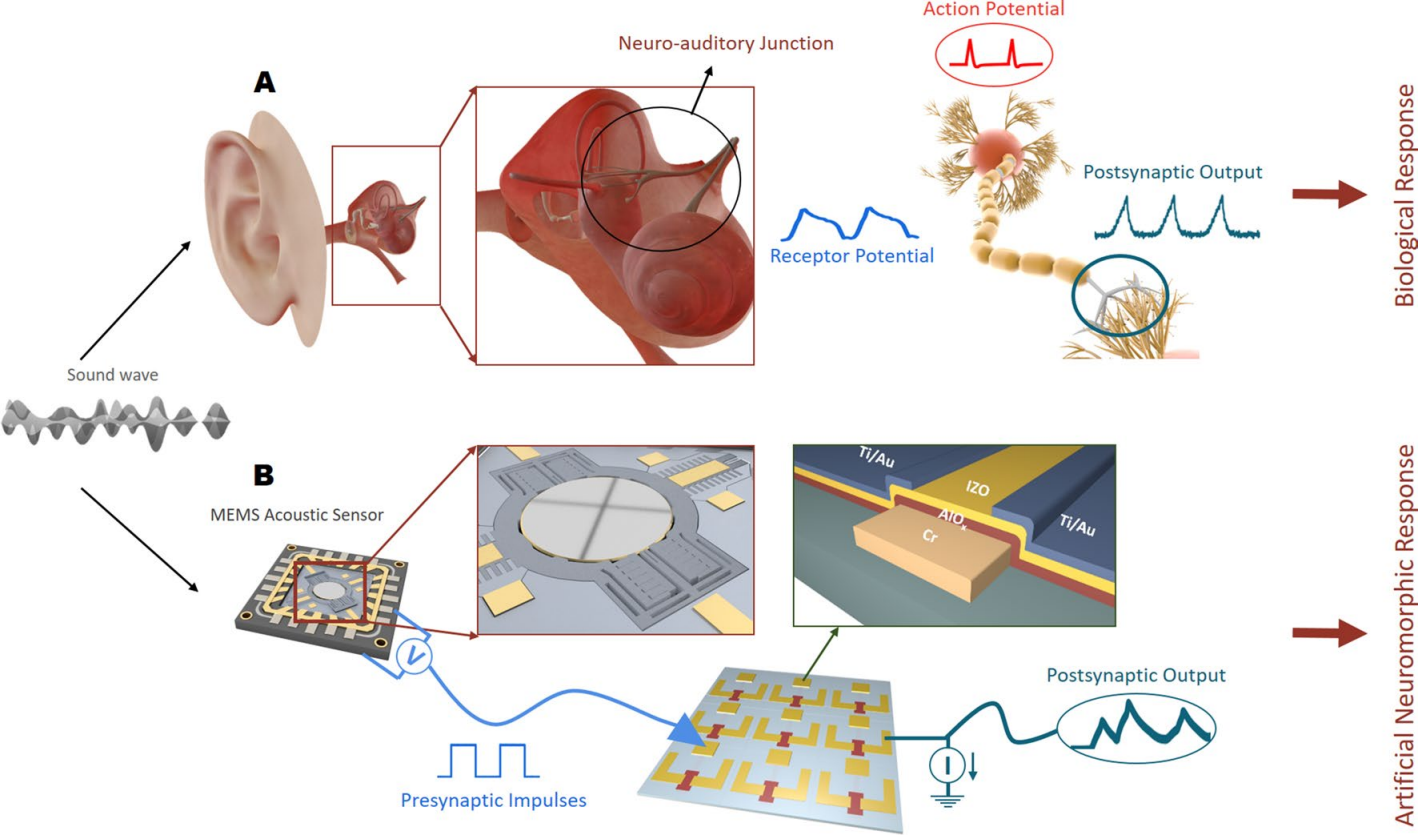
Schematic demonstration of acoustic signal processing. (**A**) Schematic presentation of the action potential creation in the human body as a result of the incoming acoustic stimulation. (**B**) Acoustic signal processing system employing synaptic transistors with printed AlO_x_ dielectrics. (Image was created by using Cinema 4D R20 from Maxon Computer GmbH https://www.maxon.net).

Figure 1 shows the schematic of the neural response in the human body to acoustic stimulation and the constructed electronic response system. In the human ear, an auditory stimulus causes vibration in the basilar membrane and the hair cells attached to it. This vibration modulates the mechanically gated ion channels resulting in an alternating current with the input stimuli frequency. Depolarization of the hair cell releases the neurotransmitters across the auditory neurons, which results in the creation of postsynaptic potentials. Upon reaching a threshold, an action potential is fired and travels toward the auditory processing part of the brain. In the electronic response system, an incoming stimulus is converted into an electrical signal via a MEMS transducer, fed into amplifiers. The amplified signal saturation creates square signals of input frequency, which is provided as presynaptic input to synaptic transistors. The transistors' output mimics the behavior of the chemical synapses.

## Results

**Figure 2:**
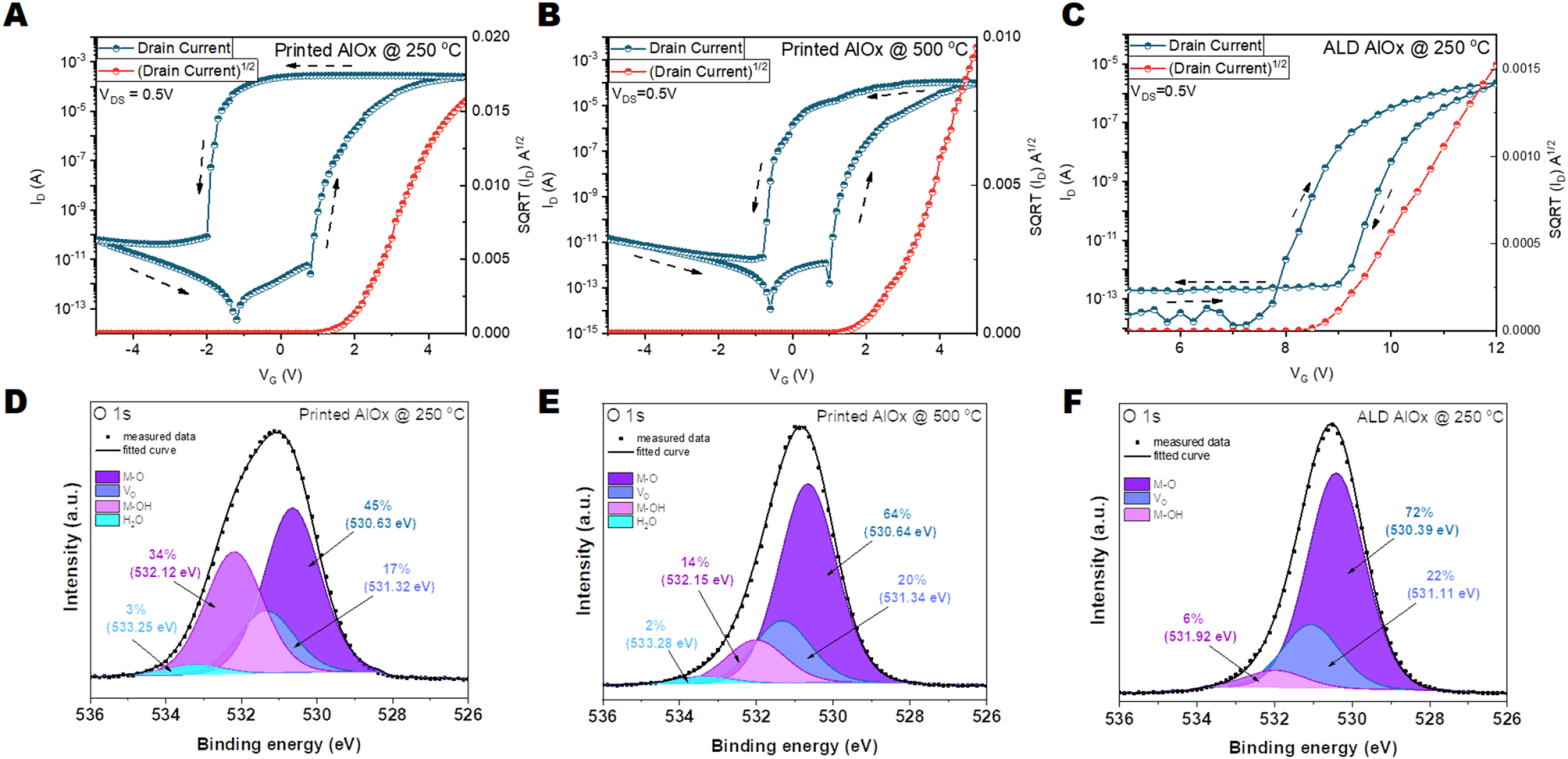
Transfer characteristics of the TFTs and XPS analysis of AlO_x_ dielectrics. Transfer characteristics of the TFTs with (**A**) printed AlO_x_ dielectrics annealed at 250 °C. (**B**) printed AlO_x_ dielectric annealed at 500 °C (**C**) ALD AlO_x_ dielectrics grown at 250 °C. XPS O 1 s peak of (**D**) printed AlO_x_ dielectric annealed at 250 °C. (**E**) printed AlO_x_ annealed at 500 °C. (**F**) ALD AlO_x_ dielectric grown at 250 °C.

Memory behavior should be present in the transistors to observe electrically activated synaptic characteristics in such devices. The existence of the memory is often reflected by hysteresis in the transfer characteristics of the TFTs^2,14^. Figure 2A shows the hysteretic behavior of the transistors employing printed AlO_x_ dielectrics and solution-processed indium zinc oxide (IZO) channels. A counterclockwise hysteresis is observed in several devices with different channel widths (W) and lengths (L), along with a uniform turn on and turn off behavior for various W/L ratios (Supplementary Fig. S1). This type of hysteresis is a sign of positive charge trapping in the dielectric at the accumulation region of the metal oxide semiconductor (MOS) structure. This results in an enhanced field effect on the semiconducting channel, resulting in the carriers' accumulation even after the gate bias is removed^27^. One striking observation is that the subthreshold slope in turn off transient (20 mV/dec) is significantly lower than the thermionic limit (60 mV/dec)^28^. This behavior suggests a ferroelectric-like charge trapping mechanism in the printed AlO_x_ dielectrics. A similar observation was reported in vacuum-based TFTs with atomic layer deposited (ALD) ultrathin AlO_x_ (5 nm) dielectrics where the charge trapping was related to the defective structure of the layer^4^. Positive charge trapping was reported to vanish for ALD AlO_x_ layers thicker than 10 nm deposited at 150 °C^4^. Here, we observe that the positive charge trapping behavior exists at three different thickness levels of printed AlO_x_ dielectrics (25 nm, 45 nm, and 60 nm) (Supplementary Fig. S1), which suppresses the gate leakage and enhances the operation voltage range. The positive charge trapping hypothesis is further supported by acquiring transfer characteristics at different gate bias ranges (Supplementary Fig. S1). The same turn-on behavior is observed in the forward sweep, regardless of the starting negative gate bias level. In contrast, for the reverse bias sweep, turn-off transient is shifted to more negative values for higher maximum gate voltage levels.

The charge trapping phenomena is further investigated by acquiring the transfer characteristics of TFTs with three different types of AlO_x_ dielectrics. Two different post-deposition temperature levels were investigated for printed layers, namely 250 °C and 500 °C. This was done to understand if the charge trapping mechanism is related to low-temperature processing conditions or is still present at high temperature processed dielectrics. ALD based AlO_x_ was used as vacuum-based reference material. AlO_x_ dielectrics printed and annealed at 250 °C (printed AlO_x_ @250 °C) (Fig. 2A), printed and annealed at 500 °C (printed AlO_x_ @500 °C) (Fig. 2B) and atomic layer deposited at 250 °C (ALD AlO_x_ @250 °C) (Fig. 2C) were implemented in TFTs, respectively. Identical semiconductor-processing conditions were maintained to have a reliable comparison among the devices (see “Methods” section). The obtained transfer characteristics show that regardless of the annealing temperature, positive charge trapping is present in the printed AlO_x_. The hysteresis window is wider in the devices with AlO_x_ dielectrics annealed at 250 °C. In contrast, a relatively narrow clockwise hysteresis (opposite direction to the printed AlO_x_ cases) is observed in ALD AlO_x_ dielectric containing transistors, which can be related to negative charge trapping at the channel/dielectric interface or within the dielectric itself^29^.

Chemical compositions of the AlO_x_ thin films fabricated and annealed at different conditions were investigated by X-ray photo spectroscopy (XPS) measurements and shown in Fig. 2D–F. Binding energies of the O 1 s peaks for the AlO_x_ films annealed at 250 °C and 500 °C (Fig. 2D,E) were deconvolved into four peaks^30–32^. In addition to the oxygen atoms with a binding energy of 530.63 ± 0.02 eV from the AlO_x_ matrix, the oxygen species with higher binding energies, such as 531.32 ± 0.02 eV and 532.12 ± 0.03 eV are assigned to oxygen vacancies and M–OH species, respectively. The small peak^32^ (2–3%) with the highest binding energy at 533.25 ± 0.04 eV is attributed to water existing in solution-processed samples. The portion of M–OH binding states (at 532.12 ± 0.03 eV) is 14% for the printed @500 °C AlO_x_ films (compare to printed @250 °C AlO_x_ films: 34%). A decreased M–OH ratio and an increased amount of M–O at high-temperature processed layers can be attributed to the conversion of more aluminum hydroxides to form aluminum oxides. N 1 s peaks of the printed layers were acquired to show that there are no remaining nitrate precursor residuals in the layers (Supplementary Fig. S2). ALD AlO_x_ film deposited at 250 °C deconvolves into three peaks, corresponding to M–O, oxygen vacancies, and M-OH species of the main O 1 s peak. This layer has the highest amount of the lattice M–O concentration, whereas no water is present in ALD AlO_x_. Existence of hydroxides and oxygen vacancies, as well as the water present in the solution-processed metal oxide dielectrics, were reported to be responsible for the anomalous charge trapping behavior^33–36^. Here we extend this hypothesis to printed AlO_x_ dielectrics annealed at two different temperature levels. As the device with the printed AlO_x_ dielectric annealed at 250 °C exhibits the widest hysteresis window relevant for memory behavior, it was investigated for its synaptic properties.

**Figure 3:**
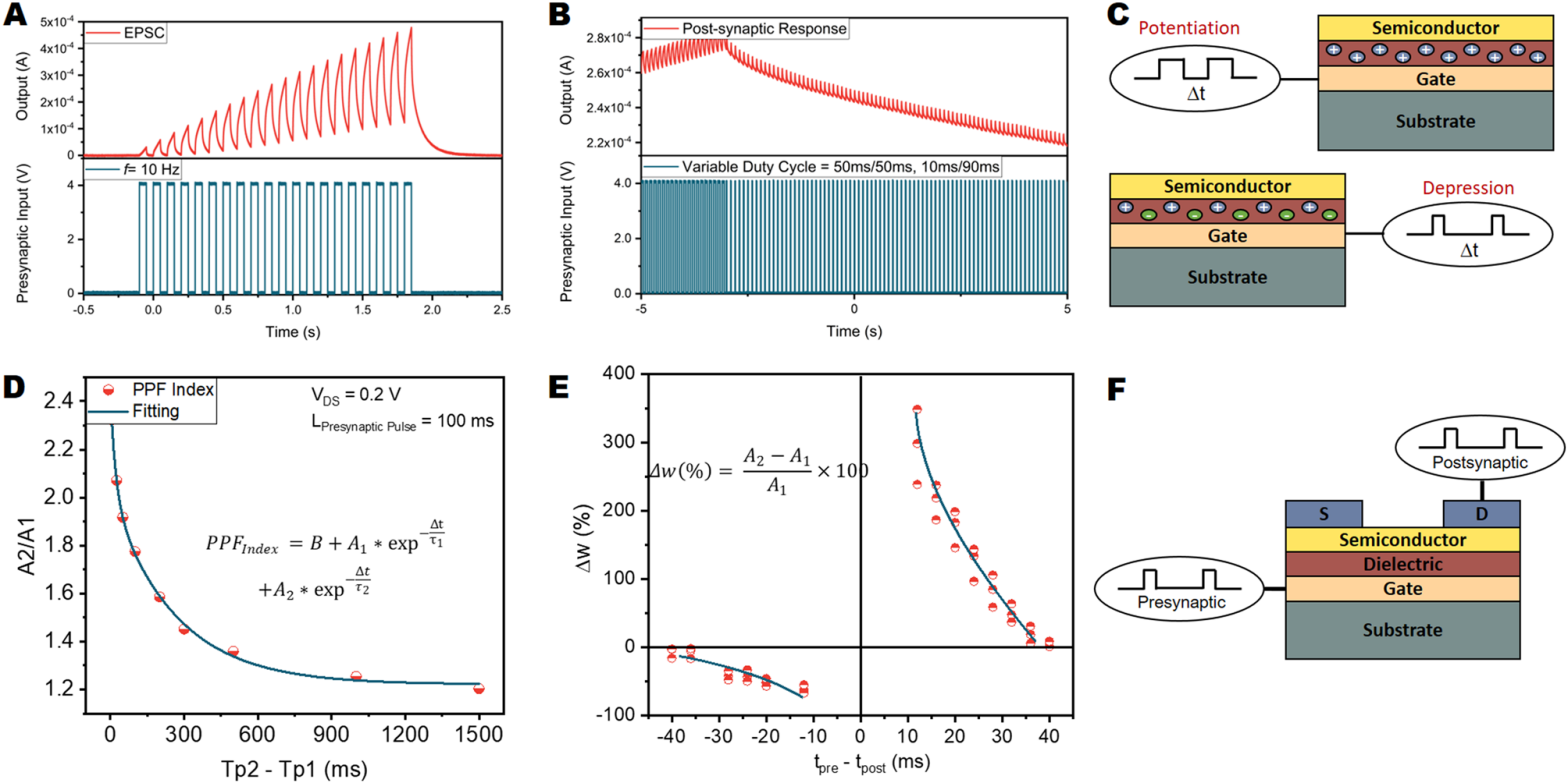
Synaptic properties of the TFTs. (**A**) excitatory postsynaptic current (EPSC) characteristics at 10 Hz operation frequency. (**B**) Potentiation and depression of the PSC obtained via modulating the duty cycle of the presynaptic input pulsing scheme. (**C**) Schematic demonstration of the trapped charges in the dielectric during potentiation and depression (Image was created by using Cinema 4D R20 from Maxon Computer GmbH https://www.maxon.net) (**D**) PPF index of the synapses with presynaptic pulse pair of 4 V magnitude and pulse length of 100 ms. (**E**) STDP characteristics of the synaptic transistors. (**F**) Schematic of the pre- and postsynaptic signal inputs of the TFTs. (Image was created by using Cinema 4D R20 from Maxon Computer GmbH https://www.maxon.net).

Synaptic behavior of TFTs was acquired by applying square-shaped pulses (presynaptic voltage) to the gate contact of TFTs and measuring their output (drain to source) currents (postsynaptic current). The excitatory synaptic response in the synaptic TFTs is reflected as an increase in the device's output current. Figure 3A shows the excitatory postsynaptic current (EPSC) at an input frequency of 10 Hz. Measurements performed at frequencies up to 50 kHz prove the devices' synaptic behavior at a wide range of frequencies (Supplementary Fig. S3).

Inhibitory synapses can be observed in various ways, one of which is to decrease the frequency of the presynaptic pulses arriving at the synaptic cleft^37^. This is demonstrated by changing the duty cycle of the input pulses arriving at the gate terminal (Fig. 3B). When the duty cycle of the presynaptic pulse is 50%, a potentiation behavior is present, followed by a depression, obtained with a decreased duty cycle of the presynaptic input (10%). During potentiation, continuous positive charge trapping in the dielectric results in an enhanced field effect on the channel layer, whereas during the depression, charge trapping is depressed due to the increased OFF gate bias period, which increases released charges from dielectric, and thus the output current decreases. (Fig. 3C).

Postsynaptic membrane potentials in the neurons possess temporal summation property^38^. When a presynaptic pulse arrives before the effect of the previously applied presynaptic action potential vanishes, their effects are summed and reflected as an increase or decrease in the postsynaptic membrane potential. The former occurs for excitatory, and the latter occurs for inhibitory presynaptic inputs. The increment in the excitatory case (ratio of the EPSC after the second pulse to EPSC of first presynaptic pulse) is named as the paired-pulse facilitation (PPF) index. Figure 3D shows the PPF index of the synaptic transistors with different time intervals between the first and second presynaptic pulses. A two-phase exponential fitting (Eq. 1) is applied to determine relaxation time constants of the PPF^5^.

1 $$ PPF = B + A_{1} \times e^{{\frac{ - \Delta t}{{\tau_{1} }}}} + A_{2} \times e^{{\frac{ - \Delta t}{{\tau_{2} }}}} . $$

The effects of the two different pulse lengths, namely 10 ms and 100 ms, were investigated in PPF index extraction to understand the frequency-related temporal behavior in the transistor (Supplementary Fig. S4). For 10 ms pulse, steady-state value is reached at a pulse spacing of 40 ms. In contrast, for the 100 ms presynaptic pulses, even at 1.5 s of an idle period, the PPF index was above 1.2, proving longer-term retention of the channel conductance.

Spike timing-dependent plasticity (STDP) is another property of the synapses, determining the strength of the synaptic potentials. It is known to be the mechanism behind the learning behavior of the human brain. The STDP behavior in the synaptic transistor is obtained by applying consecutive pulses (pulse length = 10 ms) to gate and drain contacts (Fig. 3F), as shown in Fig. 3E. An asymmetric STDP is observed with both potentiation and depression behaviors vanishing for absolute spiking time differences of 40 ms. This observation is in line with the PPF index measurements since, for the short term memory (STM), the synaptic effect disappears approximately after 40 ms.

**Figure 4:**
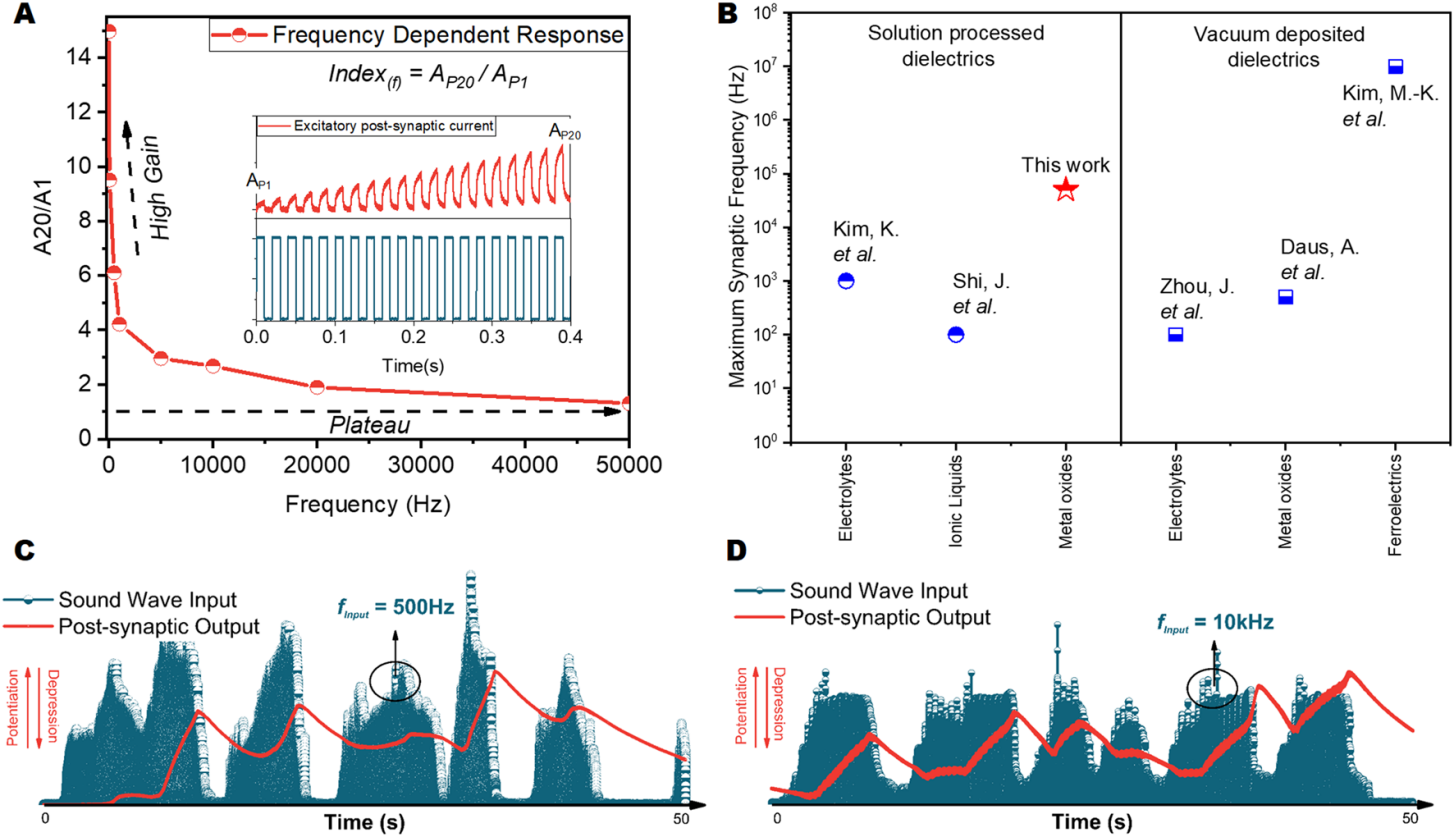
Synaptic gain at different frequencies and acoustic system response at different frequencies. (**A**) The frequency-dependent synaptic gain of the transistors. (**B**) The maximum synaptic operation frequency of transistors with different dielectric materials. Present work marks the highest operation frequency for solution-processed dielectrics. (**C**) The synaptic response of the sound system to acoustic stimulation at 500 Hz. (**D**) Synaptic response to incoming sound signals at 10 kHz.

A valuable feature of the TFTs with AlO_x_ dielectrics is the enhanced synaptic operation frequency obtained in this work up to 50 kHz. Figure 4A displays the excitatory output behavior at different frequencies. It is plotted as the amplitude ratio of the PSC of the 20th presynaptic input pulse to the PSC of the first presynaptic input pulse. Frequency related behavior of the EPSC gain is affected by mechanisms promoting positive charge trapping in the dielectric at different rates. The time required for the neutralization of the positively charged defects in the aluminum oxide dielectrics determines the frequency-dependent synaptic response of the device. Obtained capacitance frequency (C-f) characteristics from AlO_x_ dielectrics reveal a significant frequency dispersion in the low-frequency regime (< 1 kHz). In contrast, the capacitance change in the higher frequencies is quite minor (Supplementary Fig. S5). Previous studies on solution-processed aluminum oxide dielectrics indicated adsorption/desorption of the water from the environment as well as the water existing in the bulk of the dielectric, and the high amount of hydrogen as the primary sources of the counterclockwise hysteresis in the transistors originated by positive trap creation in the dielectric, along with a significant frequency dispersion in the insulator^5,36^. Investigations by Daunis et al.^36^ on solution-processed oxide transistors revealed that when water is present in the aluminum oxide, negative charge migration from dielectric to the gate under positive gate bias creates positively charged defect states in aluminum oxide, which result in field enhancement and a counterclockwise hysteresis during the turn off transient of the device. The mobile ions, such as OH^−^ and H^+^ can follow the signals below kHz frequencies, and therefore can contribute to the hysteretic behavior in the device. As the frequency increases, their contribution is expected to decrease. This is also in line with our observations in TFTs and obtained C-f characteristics. Observation of the potentiation with the positive gate bias pulses above kHz frequencies shows that positive charge trapping still exists at the high-frequency regime and therefore is expected to originate from traps with faster responses in the dielectric. Figure 4B compares the synaptic transistor operation frequency for different types of gate dielectric materials. Notably, the synaptic transistors presented in this study possess the highest operation frequency reported for any solution-processed synaptic transistor, which can enhance the application areas of such devices to acoustic signal processing.

An acoustic response system (Supplementary Fig. S6) employing TFTs with printed AlO_x_ dielectrics was built and tested with sound signals at different frequencies, to demonstrate the potential of synaptic transistors in neuro-acoustic signal processing. The response of synaptic transistors to sound signals at 500 Hz and 10 kHz are shown in Fig. 4C,D, respectively. Potentiation and depression behaviors are visible in the postsynaptic current values for the input signal above the transistor's threshold. The synaptic response of the system is also demonstrated to "Piano Sonata No. 11 in A major, K. 331—III. Rondò Alla Turca" from Wolfgang Amadeus Mozart (Supplementary video), where potentiation, depression, as well as the effects of both short term and long term memory are observed at the output signal.

## Discussion

Synaptic transistors can become fundamental elements in future applications of neuromorphic computing as well as bio-interface electronics. Printing is a convenient approach for manufacturing such synaptic transistors and related circuits because it promises low-cost roll-to-roll manufacturing of electronic and optoelectronic devices. Previously reported solution-processed synaptic transistors exhibited operation frequencies limited to 1 kHz^7,14,19,39^. The operation frequency should be improved to at least 20 kHz (upper edge of audio signals)to mimic all neuronal functions with solid-state devices and even higher for high-speed neuromorphic computing. The charge trapping phenomena in the printed AlO_x_ dielectrics results in a ferroelectric like memory behavior in transistors, which enables synaptic behavior up to 50 kHz, thus covering the whole audio frequency range. An acoustic system with synaptic responses up to 10 kHz signifies the promise of the synaptic transistors with printed oxide dielectrics for the future high-performance neuro-acoustic signal processing applications.

## Methods

### Ink preparation

AlO_x_ ink was prepared by dissolving Al(NO_3_)_3_ × 9H_2_O in 50%/50% volume 2-Methoxyethanol/Ethylene glycol. Nominal metal ion concentration was 0.4 M. Solution was stirred at 70 °C for 12 h and afterward filtered with a 0.2 µm PFTE filter. The viscosity of the ink was measured at 25 °C by a strain-controlled rheometer (Anton-Paar MCR 301) with plate/plate geometry (25 mm diameter, 0.1 mm gap) and obtained as 12 cP falling in a suitable range for inkjet printing.

IZO ink was prepared by dissolving In(NO_3_)_3_·xH_2_O and Zn(NO_3_)_2_ × 6H_2_O with a total metal ion concentration of 0.2 M (In: Zn, 7:3) in 2-Methoxyethanol. The solution was stirred at 60 °C for 12 h and filtered with a 0.2 µm PFTE filter before deposition.

### TFT fabrication

A test grade silicon wafer serves as the substrate. The substrate was sonicated in acetone (5 min), isopropanol (5 min), and DI water (5 min). Cleaned substrates underwent a buffered oxide etchant exposure to remove the native oxide from the wafers. 250 nm thick SiN_x_ was deposited via plasma-enhanced chemical vapor deposition at 150 °C to isolate the devices from the substrate. E-beam evaporated Cr (35 nm) was used as gate contact and patterned via photolithography. Dielectric layers for three different cases were deposited as follows: Printed AlO_x_ @250 °C: AlO_x_ dielectrics were inkjet printed with a resolution of 300 drops-per-inch via LP50 inkjet printer with Dimatix cartridges of 10 pl nozzle volume. Two layers of dielectric ink were printed to avoid pinholes in the insulator. Printed layers were dried at 250 °C, followed by a DUV exposure in the air for 45 min to decompose nitrates into oxides. Final annealing at 250 °C (60 min) in air ambient was applied to densify the cured layers further. Printed AlO_x_ @500 °C: Printing and drying conditions were kept the same as the Printed AlO_x_ @250 °C case. Final annealing was performed at 500 °C for 1 h. ALD AlO_x_ @250 °C: 250 cycles of trimethylaluminum pulse (60 ms) and milli-q H_2_O pulse (60 ms) with an intermittent Ar purge of 8 s between precursor pulses were applied to grow AlO_x_ at a temperature of 250 °C in a Fiji ALD reactor. The final thicknesses of the dielectric layers were measured with ellipsometry to be ~ 25 nm. Two consecutive layers of IZO ink were spin-coated with a speed of 2000 rpm for 30 s and dried at 150 °C. Similar to printed AlO_x_ @250 °C, the dried semiconductor layer underwent the DUV exposure in the air for 45 min and the last annealing step at 250 °C for 60 min. 30 nm thick semiconductor channel was photolithographically patterned and etched with a 1:121 diluted hydrochloric acid solution. Ohmic Ti/Au (5 nm/60 nm) source/drain contacts were placed via evaporation and lift-off method.

XPS measurements were performed using a Quantum2000 photo spectrometer from Physical Electronics with a monochromatic Al Kα source (1486.6 eV) operated at a base pressure below 1 × 10^−7^ Pa. The instrument's work function was calibrated to the binding energy of 83.95 eV (fwhm = 0.8 eV) for the Au 4f7/2 peak. The linearity of the energy scale was checked according to ISO 15472. An electron flood gun operated at 2.5 eV, and an ion neutralizer using Ar^+^ of approximately 1 eV were used to minimize sample charging. A short Ar^+^ sputtering (500 eV, 30 s) was performed before the measurements to remove adventitious carbon from the surface. Depth profiles were obtained with Ar + sputtering at 500 eV for 60 s per step with a material removal rate estimated to be ∼ 6 nm per sputtering step. After the third sputtering cycles, the O 1 s, as well as N 1 s peaks, were selected, and peaks were fitted with Gaussian–Lorentzian peaks and a Shirley background correction.

### Electrical characterization

DC characteristics of the TFTs were acquired in the dark with a Keithley 4200 Semiconductor parameter analyzer. Presynaptic pulses to the gate of the TFTs were provided via a signal generator (Tektronix AFG3108). Postsynaptic currents of the devices were obtained by connecting a current converter (Stanford Research Systems SR570) in series with the transistor's output. To reduce the noise in the measurements, received data was fed into a low noise amplifier and plotted via an HD oscilloscope (Teledyne LeCroy HDO6104).

AC impedance analysis of the AlO_x_ dielectrics were performed by using Paios from Fluxim AG, on MIM structures employing ITO coated glass as the bottom contact Printed AlO_x_ @250 °C as the dielectric and e-beam evaporated Al as the top contact. C-f characteristics were extracted from the obtained impedance data.

## Supplementary information

Supplementary Information.

Supplementary Video 1.

## Data Availability

Supplementary data is available in the publisher webpage. Additional supplementary information can also be obtained from corresponding authors upon request.
