# Supplementary material for: Synaptic transistors with aluminum oxide dielectrics enabling full audio frequency range signal processing
Source: Sci Rep. 2020 Oct 7;10:16664. doi: 10.1038/s41598-020-73705-w (PMC7542445; doi:10.1038/s41598-020-73705-w)
Supplement: Supplementary file 1 — Supplementary Information. [file 41598_2020_73705_MOESM1_ESM.docx]

**Supplementary Information**

**Synaptic transistors with aluminum oxide dielectrics enabling full audio frequency range signal processing**

*Sami Bolat^1+^*, Galo Torres Sevilla^1+^, Alessio Mancinelli^2^, Evgeniia Gilshtein^1^, Jordi Sastre^1^, Antonio Cabas Vidani^1^, Dominik Bachmann^1^, Ivan Shorubalko^1^, Danick Briand^2^, Ayodhya N Tiwari^1^, and Yaroslav E. Romanyuk^1^**

^1^ Empa- Swiss Federal Laboratories for Materials Science and Technology, Ueberlandstrasse 129, 8600 Dübendorf Switzerland

^2^Ecole Polytechnique Fédérale de Lausanne (EPFL), Soft Transducers Laboratory LMTS, Neuchâtel, Switzerland

^+^Equally contributing authors, *Corresponding authors

E-mail: [Yaroslav.romanyuk@empa.ch](mailto:Yaroslav.romanyuk@empa.ch), [sami.bolat@empa.ch](mailto:sami.bolat@empa.ch)


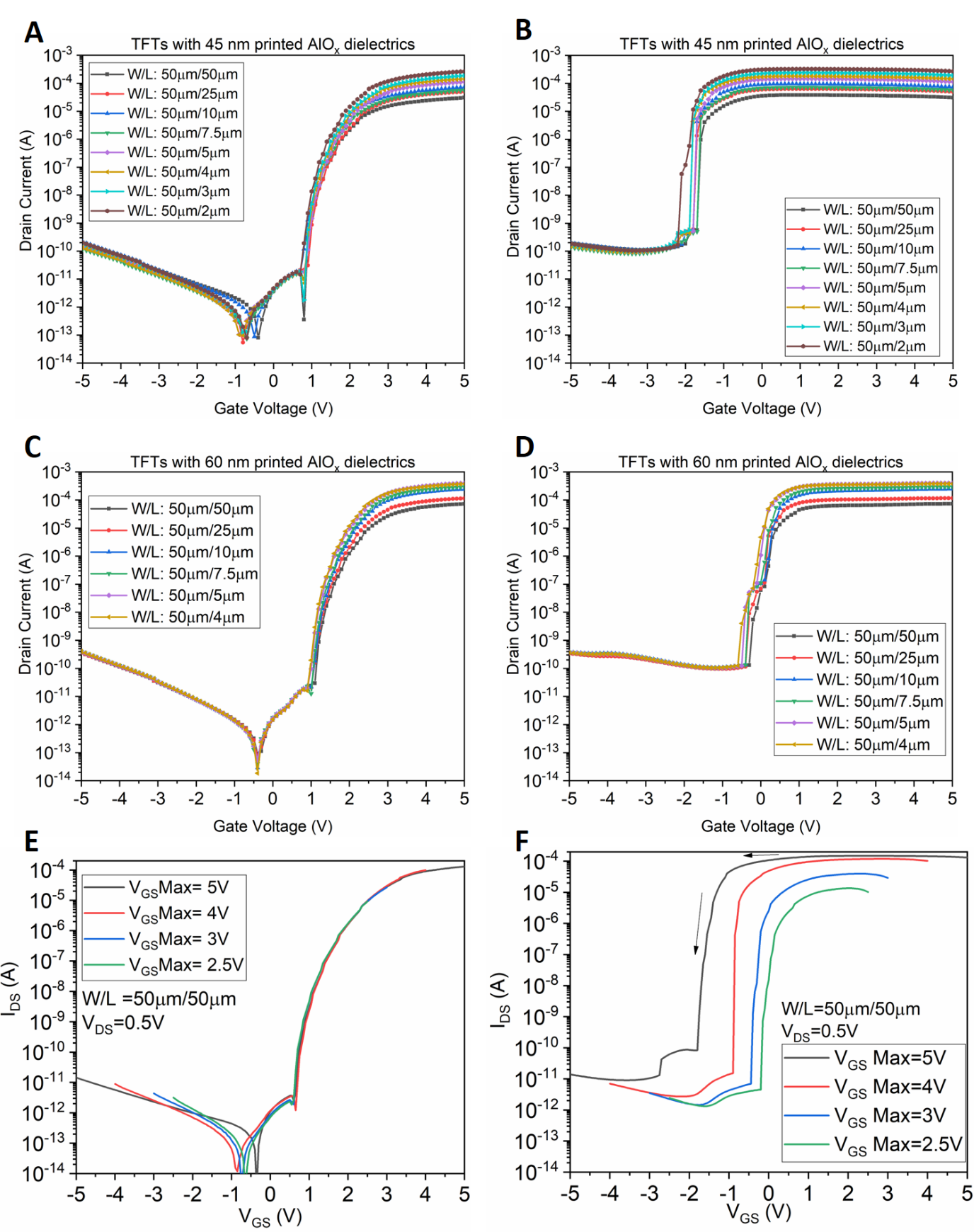


Supplementary Figure 1. **Transfer characteristics of the TFTs** (A,C) under forward gate bias sweep and (B,D) under reverse gate bias sweep. E) Effect of different gate bias sweeps on forward and F) reverse sweep transfer characteristics of the TFTs with 25 nm AlO_x_ dielectrics.

Supplementary Figure 2. **XPS N 1s peak** of AlO_x_ solution processed dielectrics annealed at 250°C (A) and 500°C (B).


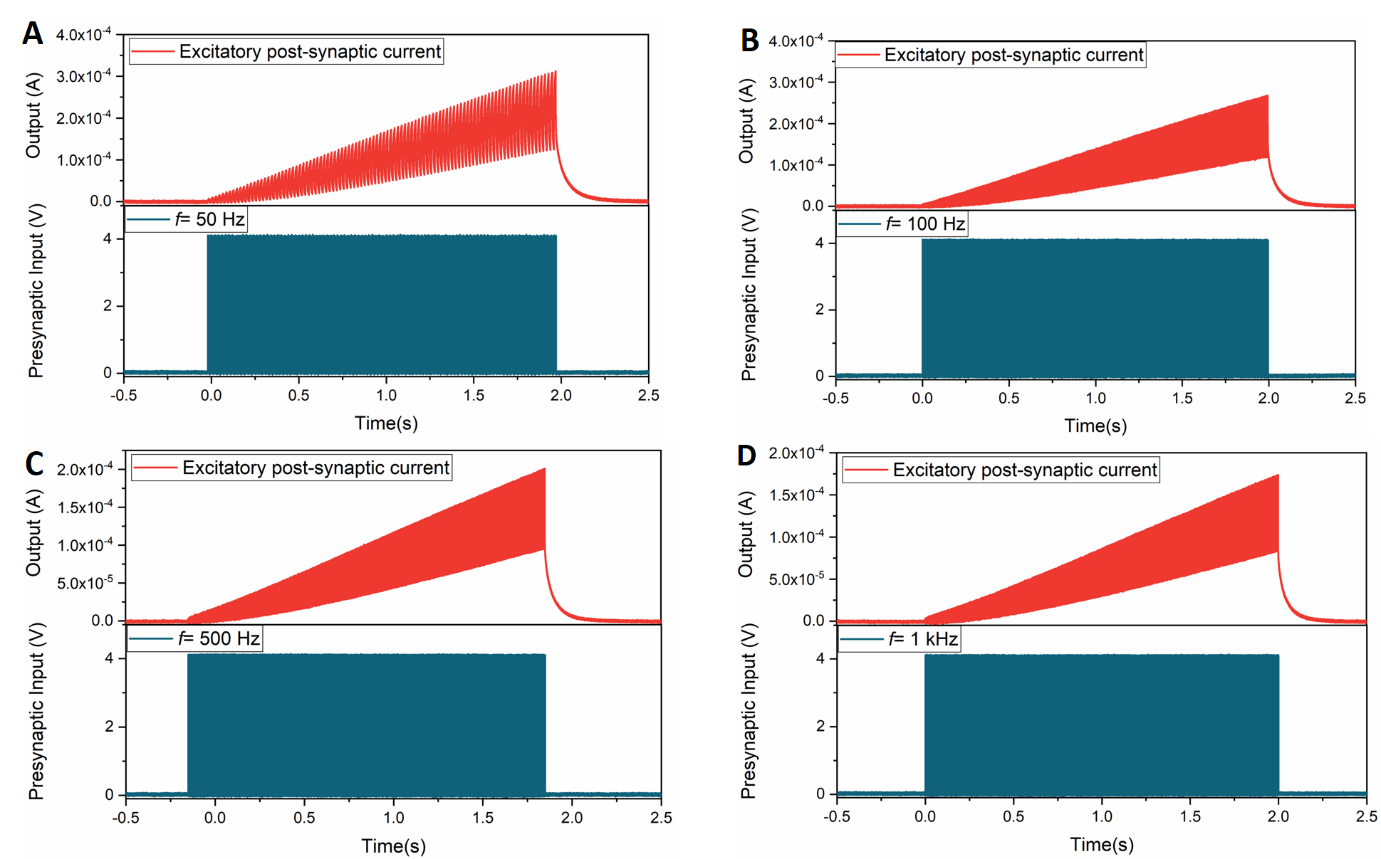


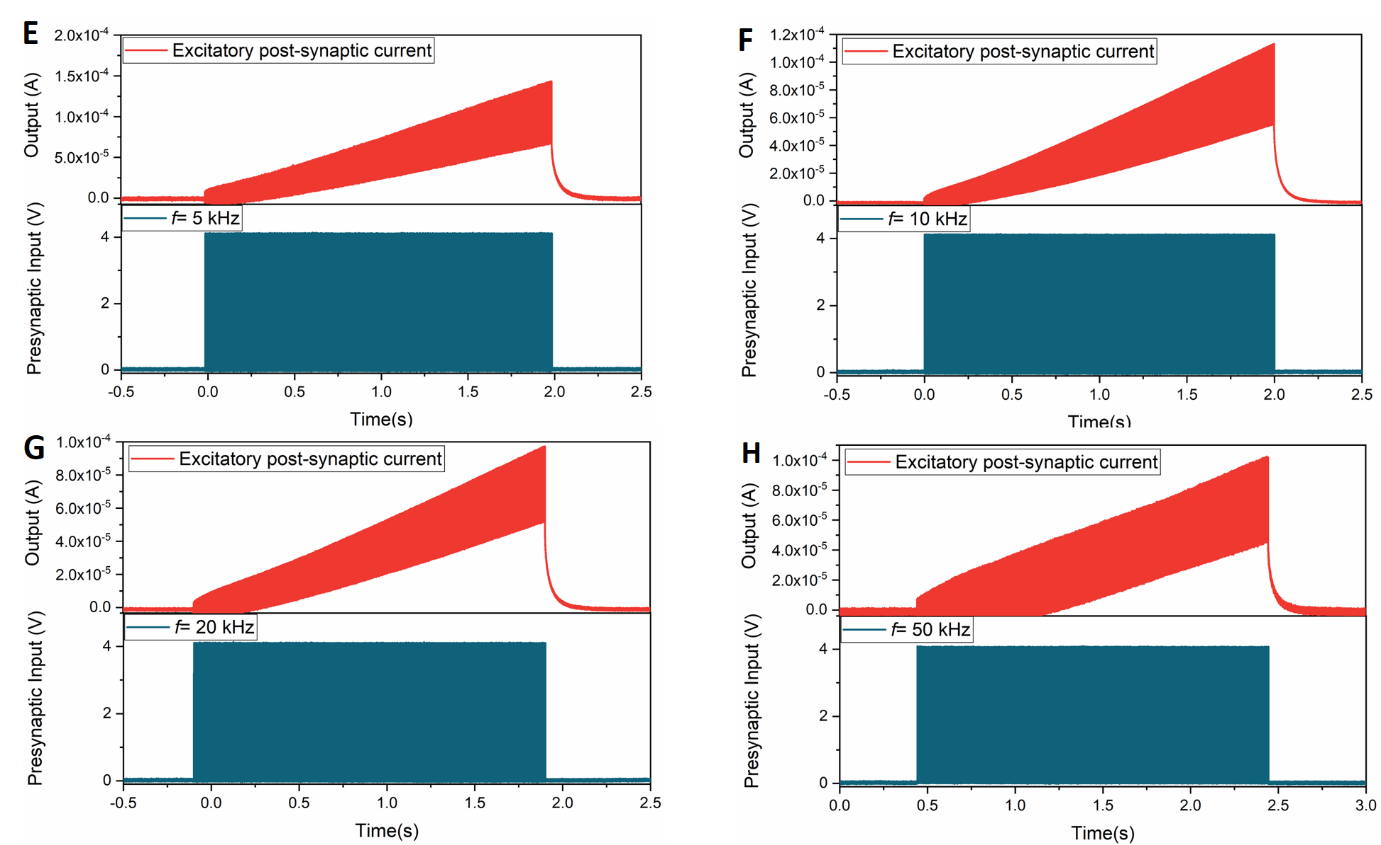


Supplementary Figure 3. **Excitatory postsynaptic currents** were acquired at various frequencies up to 50 kHz (A-H).


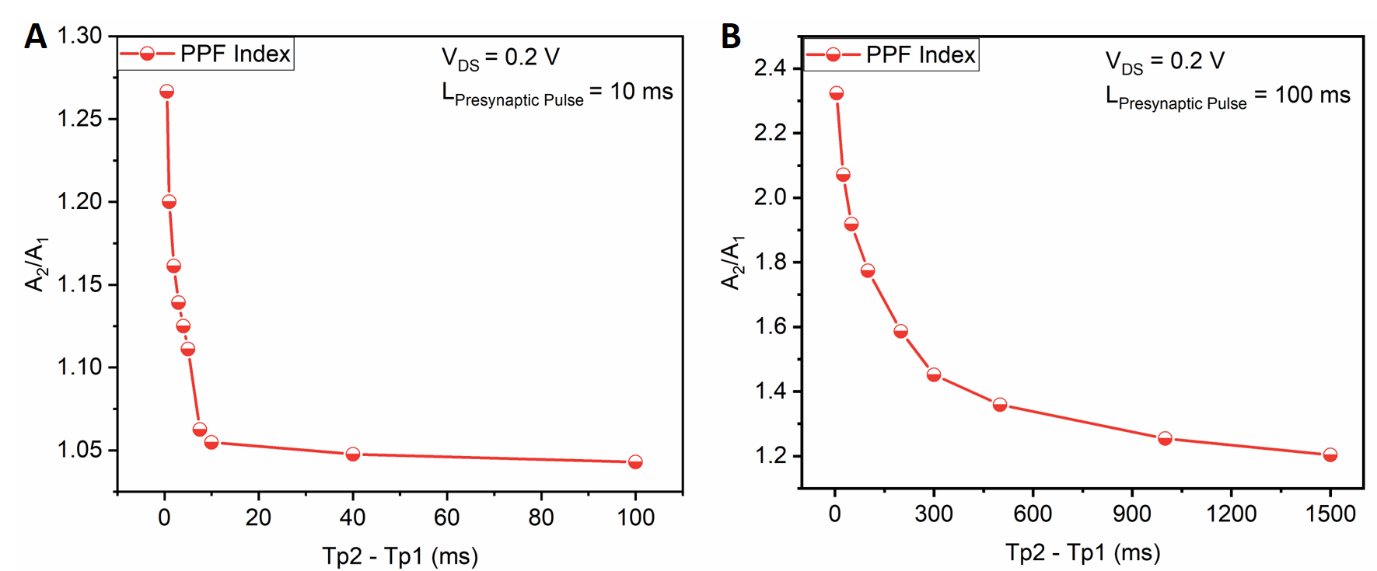


Supplementary Figure 4. **PPF index** extractions performed with two different pulse lengths show the short term memory(A) and relatively longer term memory(B) behavior of the synaptic transistors.

To investigate the temporal synaptic behavior in the transistor, effects of the two different pulse lengths were investigated in PPF index extraction, namely 10 ms and 100 ms (Figure S3). Where B in equation 1 is the steady state constant value, which is extracted as 1 in the case of 10 ms pulses, and 1.22 for 100 ms pulses. Relaxation time constants for 10 ms pulses are obtained as 0.3 ms and 4.7 ms, respectively. While for the 100 ms pulses, these values rise to 22 ms and 261 ms, respectively. For former case, steady state value is reached at a pulse spacing of 40 ms, whereas for the latter case, even at 1.5 seconds of idle period, the PPF index was above 1.2, proving a longer-term retention of the channel conductance.

Supplementary Figure 5. **C-f measurements** on printed AlOx dielectrics reveal a significant frequency dispersion in the capacitance for the frequencies below 1 kHz.


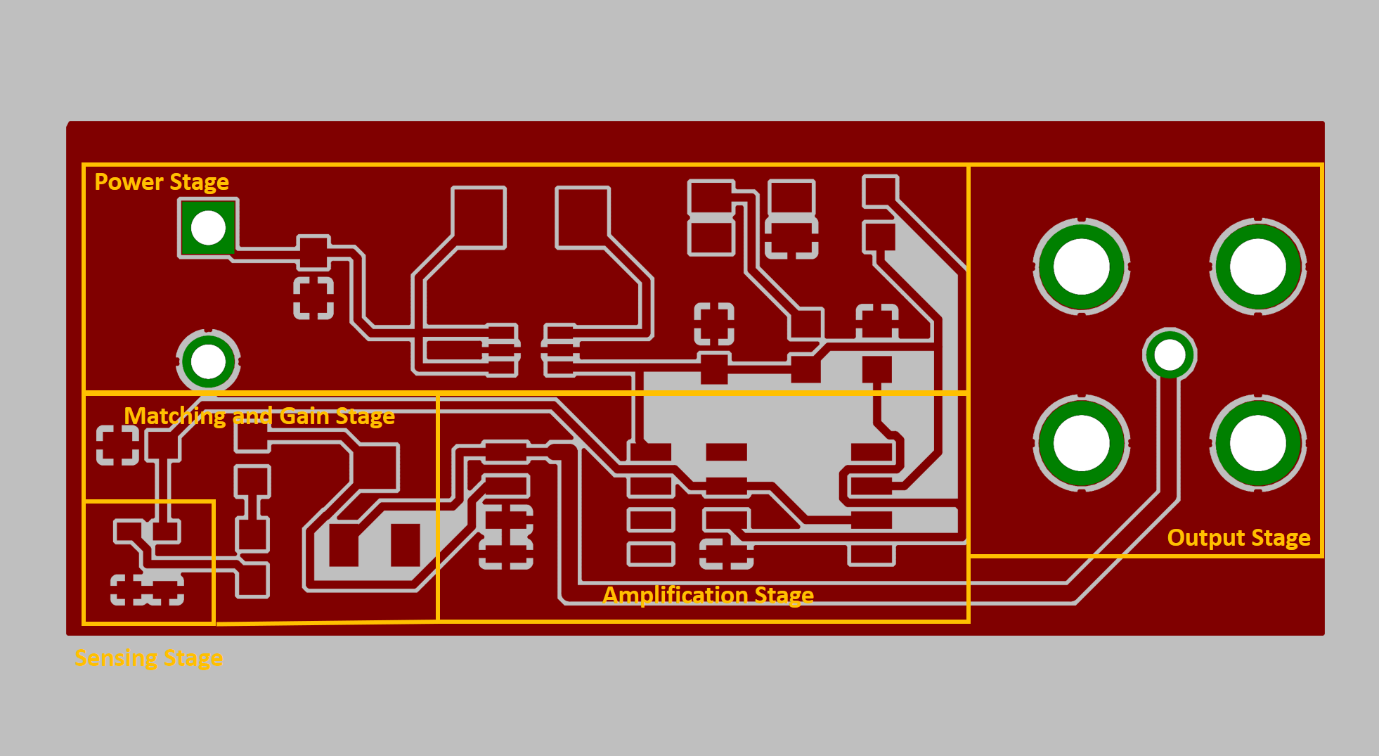


Supplementary Figure 6. **Layout of the acoustic response system**, output of which is connected to the gate terminal of the synaptic transistors with printed AlO_x_ dielectrics.

Following is the list of equipment employed at each part of the circuit:

| Power Stage | Sensing Stage | Matching Stage | Amplification Stage | Output Stage |
| --- | --- | --- | --- | --- |
| - 1. Phoenix head 2pt   2. LTC3525-5   3. ASMB-MTB0 RGB LED   4. 1 µF capacitor   5. 10 µF capacitor   6. 1 kΩ resistor   7. 10 µH inductor | - 1. SPW2430 MEMS diaphragm   2. 100 nF capacitor | - 1. 22AR100K variable resistor   2. 100 nF capacitor   3. 5 kΩ resistor | LT1006  LM358DT | 5415025 BNC Low Noise Female Connector |

Supplementary video.

Synaptic response of the system is demonstrated to "Piano Sonata No. 11 in A major, K. 331 - III. Rondò alla Turca" from Wolfgang Amadeus Mozart, where potentiation, depression, as well as the effects of both short term and long term memory are observed at the output signal.
